# Case Report: Localized hyperinflammatory response in the bulla after mosquito bites in a patient with systemic lupus erythematosus

**DOI:** 10.3389/fimmu.2026.1785094

**Published:** 2026-03-13

**Authors:** Sainan Bian, Chunmei Huang, Liping Wen

**Affiliations:** 1 Department of Allergy, Peking Union Medical College Hospital, Chinese Academy of Medical Sciences and Peking Union Medical College, Beijing, China; 2 Beijing Key Laboratory of Precision Medical for Diagnosis and Treatment of Allergic Disease, Beijing, China; 3 National Clinical Research Center for Dermatologic and Immunologic Diseases, Beijing, China; 4 Department of Laboratory Medicine, Peking Union Medical College Hospital, Chinese Academy of Medical Sciences and Peking Union Medical College, Beijing, China

**Keywords:** bullous, cytokine, hyperinflammatory response, mosquito bite, systemic lupus erythematosus

## Abstract

A patient with systemic lupus erythematosus developed localized swelling with blistering after mosquito bites. A complete blood count showed eosinophils were 1.09 × 10^9^/L (13.9%). Total IgE was more than 5,000 KU/L. Examination of the blister fluid showed very high levels of interleukin (IL)-1β, IL-2, IL-4, IL-5, IL-6, IL-8, IL-10, IL-12p70, IL-17, tumor necrosis factor (TNF)-α, interferon (IFN)-α, and IFN-γ cytokines, while these serum cytokine levels were normal. After sterile extraction of the blister fluid, the inflammatory substances were removed, and the blister gradually disappeared. This might be a special manifestation after mosquito bites in a patient with a background of autoimmune diseases. Particular attention should be paid to patients with autoimmune diseases after insect bites.

## Introduction

Allergic reactions to mosquito bites usually manifest as papular urticaria, while systemic manifestations occur in a small proportion of patients (1). Systemic lupus erythematosus (SLE) is an autoimmune disease that can involve multiple organs and is characterized by immune dysregulation (2). Patients with SLE may develop uncommon cutaneous manifestations following mosquito bites. Here, we report a case of a patient with SLE who developed localized blistering after a mosquito bite, with markedly elevated levels of inflammatory cytokines in the blister fluid, resembling a localized hyperinflammatory response.

## Case description

**Figure 1:**
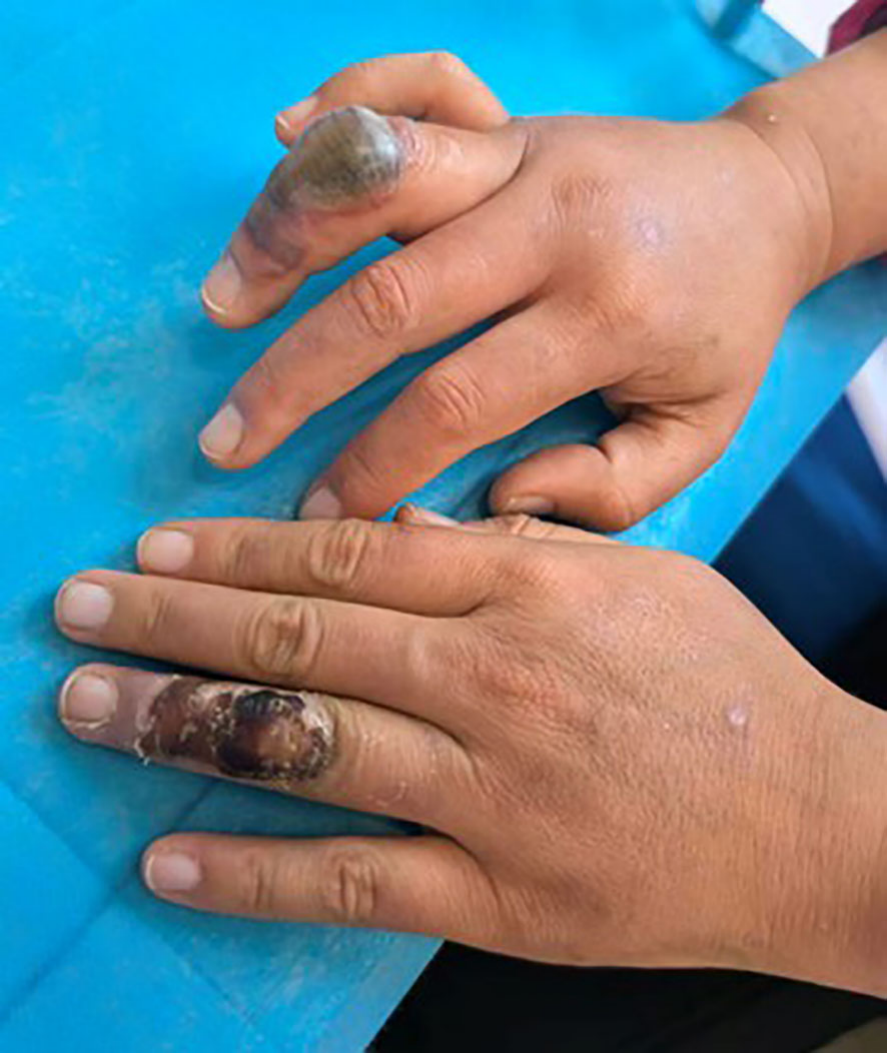
The bulla and the dusky erythematous patch following the rupture of the bulla on the fingers.

A 37-year-old female patient, newly diagnosed with SLE, presented to our department with large blisters on the fingers after mosquito bites. She had multiple lymphadenopathies and a facial rash persisting for 3 years. At our hospital’s rheumatology and immunology department, laboratory tests revealed the following results: C3 was 0.585 g/L (0.73–1.46), C4 was 0.065 g/L (0.1–0.4), IgG was 35.73 g/L (7–17), and IgG4 was within the normal range. Erythrocyte sedimentation rate (ESR) was 73 mm/h, and ANCA was negative. ANA was positive at 1:640 (speckled pattern), with anti-SSA+, anti-SSB+, anti-Scl-70+, anti-rRNP+, and anti-Ro 52 +. She was diagnosed with SLE (SLE disease activity index score was 4) and advised to initiate low-to-moderate dose glucocorticoids plus hydroxychloroquine, which she declined. Notably, she developed localized swelling with blistering after mosquito bites. Physical examination showed a 2-cm bulla on the right ring finger and a dusky erythematous patch following the rupture of a bulla on the left finger (**Figure 1**). The patient showed no fever. A complete blood count showed eosinophils were 1.09 × 10^9^/L (13.9%). Total Immunoglobulin E (IgE) was > 5,000 KU/L, Phadiatop was 3.47 PAU/L, and the insect intradermal test was negative.

**Figure 2:**
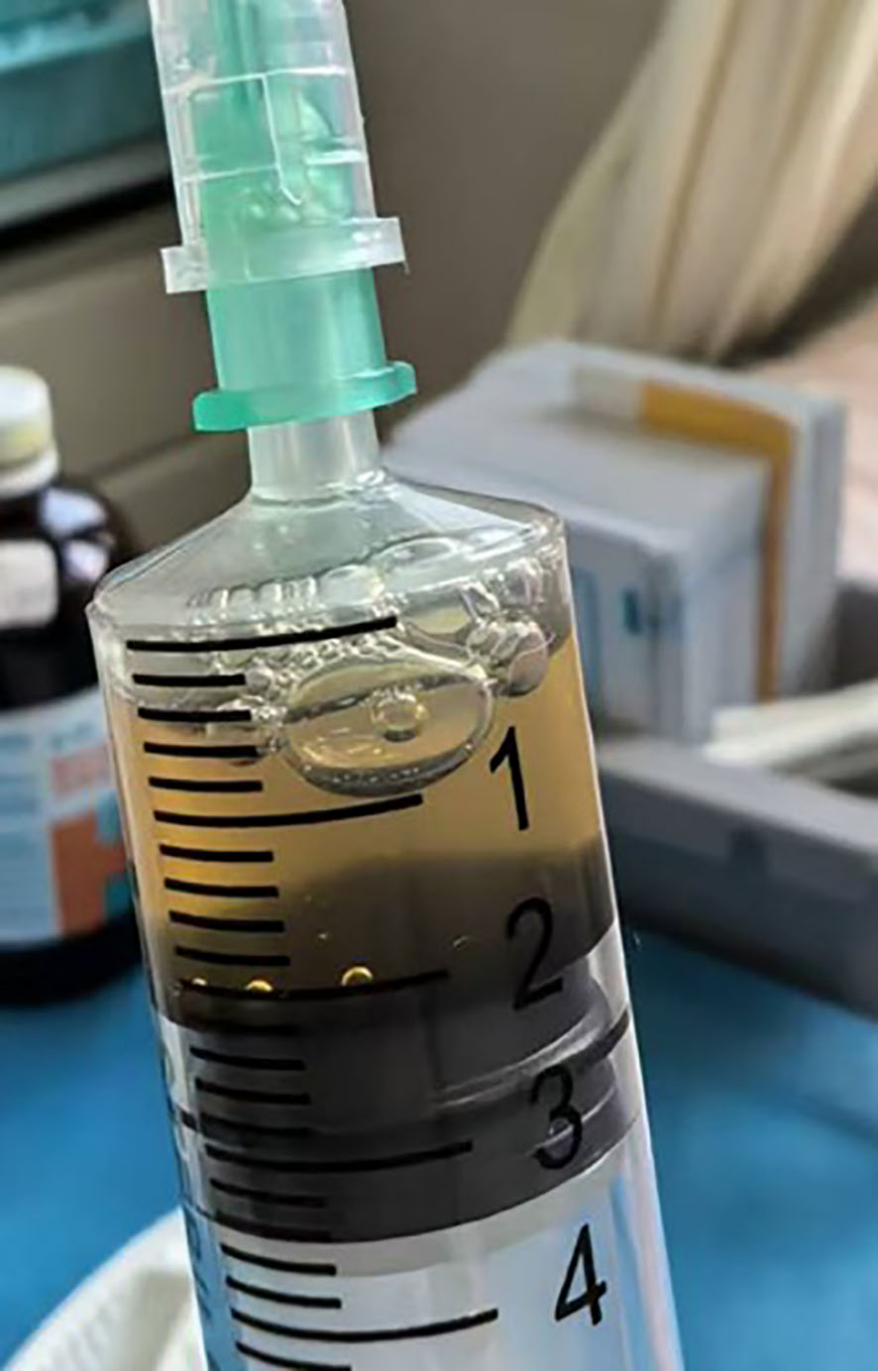
Clear and yellow blister fluid extracted from the bulla.

The doctor extracted the blister fluid, and the fluid was clear and yellow (**Figure 2**). Cytokines in both serum and blister fluid were determined using a fluorescent microsphere-based immunoassay. The assay utilized 12 types of capture microspheres with distinct fluorescence intensities, each coated with a specific interleukin antibody. Following the formation of PE-labeled sandwich complexes, interleukin concentrations were quantified by measuring the corresponding fluorescence intensity. Examination of the blister fluid showed very high levels of cytokines, while serum levels of interleukin (IL)-1β, IL-2, IL-4, IL-5, IL-6, IL-8, IL-10, IL-12, IL-17, tumor necrosis factor (TNF)-α, interferon (IFN)-α, and IFN-γ were normal. IL-6 in the blister fluid was higher than 5,000 pg/ml (the upper limit of detection); IL-8 in the blister fluid was 3,157.22 pg/ml; and IL-1β in the blister fluid was 1,098.67 pg/ml. IL-5, IL-2, IL-4, IL-10, and TNF-α in the blister fluid were 974.15, 25.43, 28.04, 20.74, and 35.67 pg/ml, respectively. As she had no fever or other systemic symptoms and the serum cytokines were normal, we considered she had a localized hyperinflammatory response in the bulla after mosquito bites. After sterile extraction of the blister fluid, the inflammatory substances were removed, and the blister gradually disappeared.

## Discussion

The common cutaneous allergic reaction to mosquitoes is papular urticaria (1). Some unusual local reactions, such as vesicular, purpuric, or bullous reactions, have been reported (3–5). One patient presented with blistering after mosquito bites with no other medical history (3). The other two patients experienced SLE flare-ups triggered by mosquito bites (4, 5).

During a mosquito bite, the insect injects substances such as anticoagulants and vasodilators through its proboscis, leading to endothelial damage and increased vascular permeability. These proteins may act as superantigens, excessively activating monocytes and macrophages (1). Given that the primary cellular sources of IL-6 include macrophages, T cells, and endothelial cells (6), excessive immune stimulation results in cytokine release characterized by burst-like secretion of IL-6, IL-8, IL-1β, and other proinflammatory mediators.

SLE is characterized by aberrant activity of the immune system, leading to variable clinical symptoms involving the skin, joints, kidneys, and central nervous system (2). The underlying immune dysregulation in SLE is likely to increase susceptibility to bullous cutaneous reactions. Furthermore, the patient’s refusal of therapy and subsequent inadequate disease control may predispose them to exaggerated inflammatory responses. Elevated eosinophil counts and IgE levels suggest a concomitant Th2-type response following the bite, while markedly increased levels of IL-1β, IL-6, and IL-8 are more consistent with a predominantly Th1-skewed hyperinflammatory response, in which Th2 pathways likely act as secondary contributors rather than primary drivers. Further studies will be needed to elucidate the precise underlying mechanisms.

The immune system is expected to recognize foreign invaders, respond proportionally to the pathogen burden, and then return to homeostasis. This response requires a balance between sufficient cytokine production to eliminate the foreign protein and avoidance of a hyperinflammatory response in which an overabundance of cytokines causes clinically significant collateral damage (6–8). In patients with SLE, immune equilibrium is dysregulated, resulting in cytokine release triggered by mosquito salivary toxins.

Previous studies have reported bullous insect-bite reactions (9). However, cytokine levels in serum or blister fluid have not been evaluated in such cases. Those studies proposed that genetic factors, atopy, and immunodeficiency may contribute to bullous formation (9). In the present study, we measured cytokine levels both in serum and blister fluid, which, to our knowledge, represents the first report on the cytokine profile of blister fluid in this condition. Our findings expand the current understanding of the mechanisms underlying blister formation and inflammatory responses following mosquito bites.

In conclusion, we report a patient with SLE who developed a localized hyperinflammatory response after mosquito bites, which might represent a special manifestation of mosquito bites. Attention should be paid to whether patients with autoimmune diseases exhibit distinctive clinical manifestations following mosquito bites.

## Data Availability

The raw data supporting the conclusions of this article will be made available by the authors, without undue reservation.
